# Recrystallization Behavior of a Mg-5Zn Alloy Influenced by Minor SiC_p_ during Hot Compression

**DOI:** 10.3390/ma15238498

**Published:** 2022-11-29

**Authors:** Quanxin Shi, Cuiju Wang, Kunkun Deng, Kaibo Nie, Wei Liang

**Affiliations:** 1 Shanxi Key Laboratory of Advanced Magnesium-Based Materials, College of Materials Science and Engineering, Taiyuan University of Technology, Taiyuan 030024, China; 2 Instrumental Analysis Center, Taiyuan University of Technology, Taiyuan 030024, China

**Keywords:** Mg alloy, DRX mechanism, precipitation, compression

## Abstract

The influence of minor SiC_p_ on the dynamic recrystallization (DRX) and dynamic precipitation behaviors of the Mg-5Zn matrix were investigated through the hot compression test. The results showed that the addition of SiC_p_ improved the DRXed ratio of Mg-5Zn matrix, but the recrystallized grains in 1 vol.% 5 μm SiC_p_/Mg-5Zn material were mainly formed by the “bulging” nucleation of the grain boundary at a low compressive strain (~0.05, ~0.1 and ~0.35), and PDZ (particle deformation zone) around SiC_p_ had little effect on the recrystallization nucleation. However, the fine recrystallized grains appeared around the particles when the compressive strain reached ~0.7, which was attributed to the promotion effect of PDZ on recrystallization nucleation. This shows that PDZ around particles can promote DRX nucleation under large strain. Meanwhile, compared to the Mg-5Zn alloy, the volume fraction and size of the secondary phase in the SiC_p_/Mg-5Zn material increased due to the influence of SiC_p_ on the recrystallization behavior of Mg-5Zn matrix.

## 1. Introduction

As the lightest structural metal material in nature, magnesium (Mg) alloys have been widely used in the automotive, aerospace and other fields [1,2]. However, the lower plasticity and strength at room temperature limits their application [3,4].

Grain refinement is an important means to improve the comprehensive mechanical properties of materials. It is a common and effective measure to refine grains by the plastic deformation of metal materials [5]. Dynamic recrystallization (DRX) often occurs in the process of plastic deformation, which can achieve the effect of grain refinement, leading to the improvement of mechanical properties [3,6,7].

In recent years, it has been found that the mechanical properties of Mg alloys can be significantly improved by the addition of heterogeneous particles [8,9]. Nie et al. [10] revealed that the yield strength (YS) and tensile strength (UTS) of TiC/Mg-4Zn-0.5Ca composites processed by combining multidirectional forging and extrusion reached ~404 MPa and ~450.3 MPa. Shi et al. [9] found that the mechanical properties of pure Mg were significantly improved by adding SiC_p_ into pure Mg. Sun et al. [11] showed that the superior YS (371.5 MPa) and UTS (443.9 MPa) of SiC_p_/AZ91 composite were obtained after extrusion deformation with a low temperature and slow strain rate. Moreover, Wang et al. [12] reported that the elastic modulus and tensile strength both increased with an improvement in the volume fraction of SiC_p_ from 5% to 15%, but a decrease in the elongation. The improvement in the mechanical properties of the particle-reinforced Mg matrix composites is attributed to the influence of particles on the microstructure of Mg matrix. 

It is generally believed that the particle deformation zone (PDZ) with a high dislocation density and larger misorientation gradient is formed around particles during deformation due to the deformation mismatch between particles and the Mg matrix, which can promote DRX nucleation and refine grains [13]. Wu et al. [14] discovered that the grain sizes of AZ91 matrix near SiC_p_ were smaller than those far away from SiC_p_ when a SiC_p_/AZ91 composite is forged. Wang et al. [15] found that PDZs were formed near particles during hot compression and DRX preferentially started near particles due to PSN (particle-stimulated nucleation) in PDZ. All the above studies indicated that the addition of particles in Mg alloys leads to the formation of PDZ around the particles during deformation, which promotes the DRX nucleation of Mg matrix. 

The current research on the influence of heterogeneous particles on the DRX behavior of Mg alloys has mainly focused on materials with a large volume fraction (more than 5 vol.%) of particles and large plastic strain, such as forging and extrusion. However, the large volume fraction of particle leads to the overlapping of PDZ and obvious dislocation density increases, which significantly improves the influence of PDZ on the DRX nucleation [15]. Meanwhile, the microstructure observed in PDZ is usually that after DRX for particle-reinforced Mg matrix materials with large plastic deformation. 

Therefore, in order to clearly study the effect of single PDZ on DRX and dynamic precipitation behaviors of Mg alloy during hot deformation, the Mg-5Zn alloy and 1 vol.% 5 μm SiC_p_/Mg-5Zn material were prepared in this paper, and the distributed compression tests were performed. The microstructures of Mg-5Zn alloy and SiC_p_/Mg-5Zn material under different compression strains (~0.05, ~0.1, ~0.35 and ~0.7) were analyzed, and the influence mechanism of particles on the DRX and precipitation behaviors of Mg alloys was discussed. 

## 2. Experimental Procedures

In this work, the Mg-5Zn alloy was used as the matrix alloy, which was prepared by melting at 750 °C, and the whole process was carried out under the protection of mixed gas of CO_2_ and SF_6_. SiC_p_ was selected as the reinforcement, and the size and volume fraction of SiC_p_ were 5 μm and 1 vol.%, respectively. The SiC_p_/Mg-5Zn material was fabricated by semisolid stirring-assisted ultrasonic treatment under the protection of a CO_2_ and SF_6_ gas mixture. The specific preparation process of SiC_p_/Mg-5Zn material was as follows [16]: firstly, the Mg-5Zn alloy was melted in a crucible at 750 °C, then the temperature was decreased to the semi-solid temperature (~640 °C), and the SiC_p_ preheated at 600 °C was added into the Mg-5Zn alloy quickly and evenly during stirring. After stirring for a certain period, the temperature was improved to 705 °C, and ultrasonic treatment was carried out for about 10 min. After that, the molten SiC_p_/Mg-5Zn material was poured into the mould which had been preheated to 400 °C, and then it was solidified under a pressure of 100 MPa.

The ingots of Mg-5Zn alloy and SiC_p_/Mg-5Zn material prepared for compression test were machined to cylindrical specimens of 10 mm in diameter and 15 mm in height, and the solution treatment was carried out before compression (320 °C for 8 h and 430 °C for 12 h). Compression tests were carried out on a Gleeble-3800 thermal simulator at the temperature of 573 K and a compression speed of 0.01 s^−1^. The compression strains were ~0.05, ~0.1, ~0.35 and ~0.7, respectively, and the water quenching treatment was carried out immediately after compression. During the compression tests, the samples were heated to the target temperature and kept for 3 min to obtain a uniform deformation condition.

The microstructure of the Mg-5Zn alloy and SiC_p_/Mg-5Zn material were characterized by optical microscopy (OM: Olympus D11, Leica, Germany), scanning electron microscopy (SEM: TESCAN MIRA 3 LMH, Brno, Czech Republic), electron back scattered diffraction (EBSD: Oxford, UK) and transmission electron microscopy (TEM: JEM-2100F, JEOL, Tokyo, Japan). The samples of OM, SEM and EBSD were cut along the compression direction, and the samples for OM and SEM were etched in a solution of oxalic acid (4 g oxalic acid + 100 mL H_2_O) to recognize the grain boundaries and the secondary phases. The DRXed grains and secondary phase in Mg-5Zn alloy and SiC_p_/Mg-5Zn material after compression were analyzed by Image Pro-Plus software. The secondary phase composition in the materials was determined by XRD (CuKα radiation, speed of 5°/min, Rigaku, Tokyo, Japan) and EDS (Oxford, UK). The EBSD samples were prepared by grinding on the different emery papers, then electro-polished with the 5% perchloric acid solution at about −30 °C for 1 min. The data from the EBSD were analyzed by using the channel 5 software. The TEM samples were prepared by electro-polishing and observed under the condition of an accelerating voltage of 200 KV. Moreover, the macro-texture of the as-compressed Mg-5Zn and SiC_p_/Mg-5Zn material was measured by neutron diffraction (1.68 Å, Ge monochromator, Helmholtz-Zentrum). 

## 3. Results

### 3.1. Microstructure of As-Homogenized Materials

**Figure 1:**
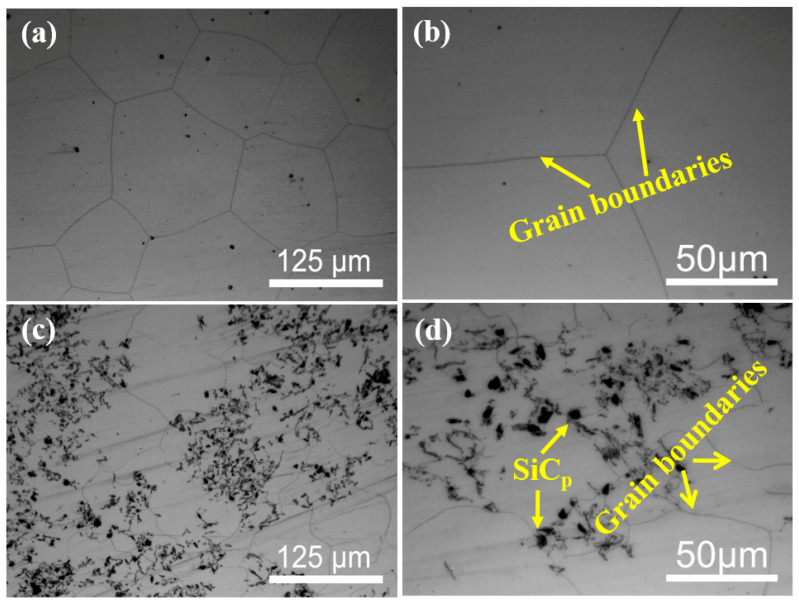
OM microstructure of as-homogenized (**a**,**b**) Mg-5Zn alloy and (**c**,**d**) SiC_p_/Mg-5Zn material.

Figure 1 shows the optical microstructure (OM) maps of as-homogenized Mg-5Zn alloy and SiC_p_/Mg-5Zn material. It can be seen from Figure 1 that there was no obvious secondary phase in Mg-5Zn alloy and SiC_p_/Mg-5Zn material after homogenization treatment, indicating that the secondary phases in the as-casted Mg-5Zn alloy and SiC_p_/Mg-5Zn material were almost completely dissolved into the matrix alloy. The average grains size measured by Image Pro-Plus software was ~114.1 μm and ~56.3 μm for Mg-5Zn alloy and SiC_p_/Mg-5Zn, respectively. 

### 3.2. DRX Behavior

#### 3.2.1. Mg-5Zn Alloy

In order to study the influence of minor particles on the DRX behavior of Mg-5Zn alloy, the distributed high-temperature compression tests of Mg-5Zn alloy were firstly performed in this section, and the microstructure under different compression strains was analyzed.

**Figure 2:**
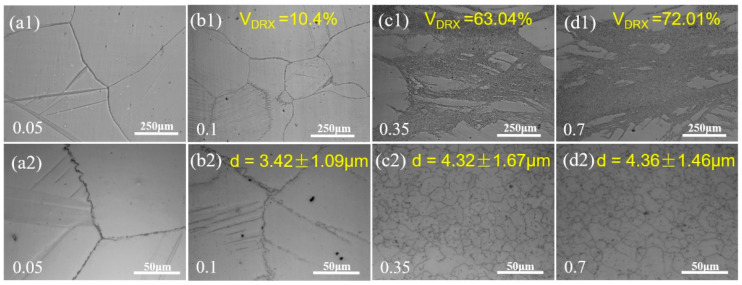
OM microstructure of Mg-5Zn alloy with different compression strains: (**a1**,**a2**) 0.05, (**b1**,**b2**) 0.1, (**c1**,**c2**) 0.35 and (**d1**,**d2**) 0.7.

The OM microstructure of Mg-5Zn alloy under different compression strains is exhibited in Figure 2. It can be seen that the original grain boundaries of Mg-5Zn alloy bent and bulged to the adjacent grains when the compression strain was ~0.05. Meanwhile, the twin microstructure was found in the grains.

As the compression strain increased to ~0.1, it can be seen that there were fine DRXed grains at the original grain boundaries, but DRX was incomplete. At the same time, the bulging phenomenon of the original grain boundaries of Mg-5Zn alloy was more obvious.

The bulging of the grain boundary for the Mg alloy was mainly caused by the non-uniformity of deformation [17,18]. The grains with a soft orientation are prone to slip during plastic deformation, while the grains with a hard orientation are difficult to start to produce plastic deformation. The dislocation density in grains with large deformation degree is high and the storage energy is large, while the dislocation density in grains with a small deformation degree is low and the storage energy is low [17,18]. The grain boundaries of two adjacent grains slide towards the grains with large deformation degree under the effect of deformation storage energy, and the storage energy in the area where the grain boundaries slip decreases, forming a low distortion area or a distortion free area [17,18]. The DRX core will be formed when the grain boundary slip area reaches a certain size, which is called the DRX “bulging” nucleation mechanism [17,18]. The average DRXed grain size and volume fraction of the Mg-5Zn alloy were ~3.4 μm and ~10.4%, respectively, when the compression strain was ~0.1.

In addition, the twins were observed in the Mg-5Zn alloy at the initial stage of deformation (~0.05 and ~0.1). The main reason is that the number of starting slip systems is small in the early stage of the plastic deformation of Mg alloy, which will lead to twinning in some areas due to stress concentration [18]. Generally, the twin type in a Mg alloy can be determined by the misorientation angle between the twin and the matrix. The misorientation angles of ~86.3°, ~56.2° and ~37.5° represent (10$\bar{1}$2) tensile twins, (10$\bar{1}$1) compressive twins and (10$\bar{1}$1)–(10$\bar{1}$2) double twin, respectively [19].

**Figure 3:**
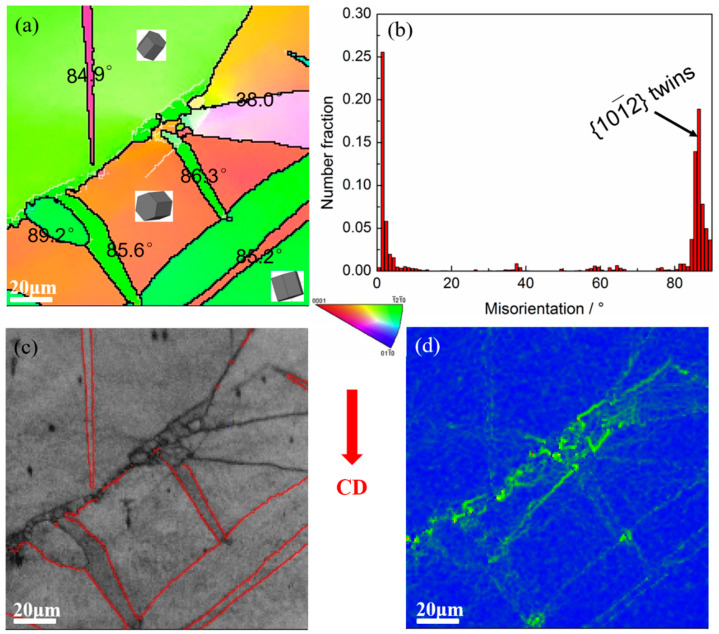
EBSD results of the Mg-5Zn alloy with a compression strain of 0.1: (**a**) IPF map, (**b**) misorientation map, (**c**) BC map and (**d**) KAM map.

Therefore, EBSD technology was used to test the Mg-5Zn alloy with a compression strain of ~0.1, as shown in Figure 3. As shown in Figure 3a–c, the twins formed in Mg-5Zn alloy were mainly (10$\bar{1}$2) tensile twins. Moreover, DRXed grains can be seen at the intersection of twins and grain boundaries, as shown in Figure 3c. The twins often terminated at the grain boundaries owing to the grain boundaries hindering the twinning. Meanwhile, the movement of dislocations was hindered by the grain boundaries and twin boundaries, leading to the stacking of dislocations and the increase in dislocation density, which promoted the nucleation of DRXed grains. Nie et al. [20] introduced a large number of tensile twins into the AZ31 alloy by bending and straightening and found obvious DRXed grains at the intersection of twins and grain boundaries.

The driving force of DRXed grain nucleation mainly comes from the strain energy, and the strain energy is different at different positions in Mg alloy, resulting in a varied DRX nucleation rate [21]. It can be seen from the KAM (Kernel Average Misorientation) map that a higher local strain energy was generated at grain boundaries, especially at the intersection of grain boundaries and twins, as shown in Figure 3d. Therefore, DRX nucleation occurs preferentially at the twins and grain boundaries.

The deformation storage energy in Mg-5Zn alloy increased with an increase in the compression strain to ~0.35, leading to an enhancement in the driving force of recrystallization, which significantly improved the quantity of the recrystallized grains in the Mg-5Zn alloy, as shown in Figure 2c. Furthermore, there were also large deformed grains in addition to the fine recrystallized grains in the Mg-5Zn alloy. The DRXed grain size and DRXed ratio were ~4.3 μm and ~63%, respectively. However, compared with Figure 2b1, it can be seen that the (10$\bar{1}$2) tensile twins formed at the initial stage of deformation almost disappeared with an improvement in the compression strain from ~0.1 to ~0.35.

**Figure 4:**
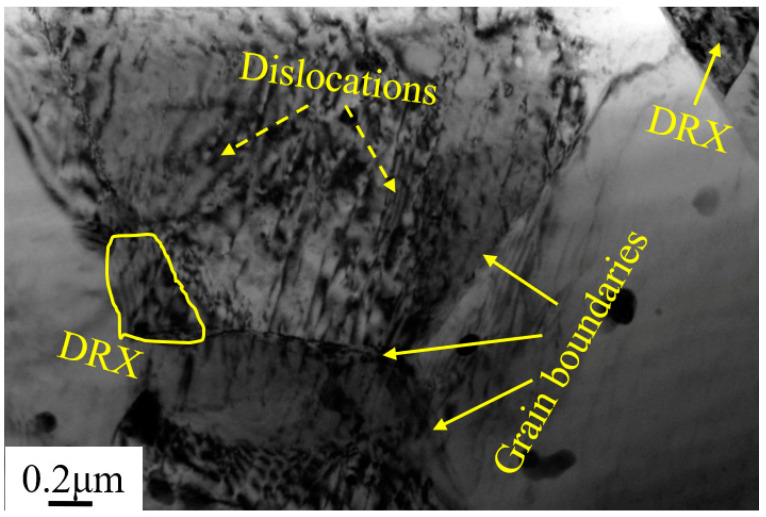
TEM microstructure of the Mg-5Zn alloy with a compression strain of 0.35.

Figure 4 shows the TEM microstructure of Mg-5Zn alloy with a compression strain of ~0.35. It can be seen that high density dislocations were formed in the grains, and the fine DRXed grain can also be seen at the original grain boundary, which further confirms that the bulging of grain boundary is the main nucleation mechanism of DRXed grain in the Mg-5Zn alloy.

As the compression strain of the Mg-5Zn alloy increased to ~0.7, the large deformed grains obviously disappeared, while the amount of fine DRXed grains increased. The average DRXed grain size and volume fraction were ~4.4 μm and ~72%, respectively, as shown in Figure 2d1,d2.

Therefore, it can be concluded from the above analysis that the dislocation density in the grains of Mg-5Zn alloy varies due to the non-uniformity of grain deformation during compression, which makes the bulging nucleation of original grain boundary under the drive of distortion energy, forming fine DRXed grains. In addition, the twins formed in the Mg-5Zn alloy at the initial stage of compression also play a role in promoting DRX nucleation. Hence, the bulging nucleation of grain boundary and twin-induced recrystallization nucleation are the main nucleation mechanisms of Mg-5Zn alloy. The average DRXed grain size and DRXed ratio in the Mg-5Zn alloy under different strains were statistically analyzed by Image Pro-Plus (6.0) software. It was found that the average DRXed grain size and DRXed ratio in the Mg-5Zn alloy increased with the increment of compression strain.

#### 3.2.2. SiC_p_/Mg-5Zn Material

**Figure 5:**
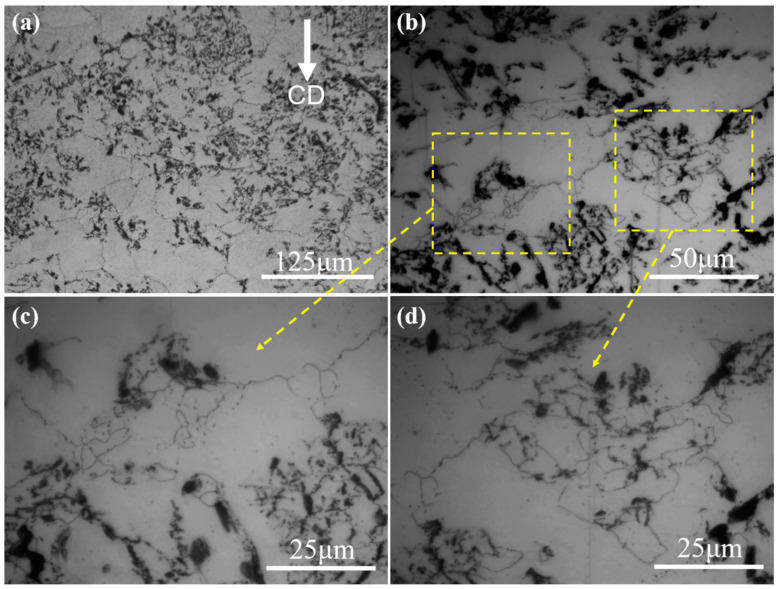
OM microstructure of the SiC_p_/Mg-5Zn material with a compression strain of 0.05: (**a**,**b**) low magnification and (**c**,**d**) high magnification.

Figure 5 displays the OM microstructure of SiC_p_/Mg-5Zn material with a compression strain of ~0.05. Compared with the Mg-5Zn alloy (Figure 2a1,a2), it can be seen that the SiC_p_/Mg-5Zn material has obvious DRXed grains, and the DRXed grains were mainly distributed along the original grain boundaries. However, no DRXed grains were found in the Mg-5Zn alloy when the compression strain was ~0.05. Thus, it can be known that the addition of SiC_p_ promotes the DRX nucleation of the Mg-5Zn matrix. Similar to the Mg-5Zn alloy, the bulging of grain boundary was also found in the SiC_p_/Mg-5Zn material, as shown in Figure 5. It can be concluded that the bulging of the grain boundary is also a nucleation mechanism of DRXed grains of the SiC_p_/Mg-5Zn material [17]. The average DRXed grain size and volume fraction of SiC_p_/Mg-5Zn were ~3.6 μm and ~8.6% when the compression strain was ~0.05.

In addition, no twins were found in the SiC_p_/Mg-5Zn material when the compression strain was ~0.05. This is inconsistent with the microstructure of a Mg-5Zn alloy. It manifests that the addition of SiC_p_ in Mg-5Zn alloy may inhibit the formation of twins, which may be related to the SiC_p_ and smaller initial grain size in SiC_p_/Mg-5Zn material. On the one hand, the bearing capacity of SiC_p_/Mg-5Zn material increased due to the fact that SiC_p_ has a higher strength and hardness than the Mg alloy matrix, which makes it difficult for SiC_p_/Mg-5Zn material to produce twins after compression. On the other hand, the addition of SiC_p_ in Mg-5Zn alloy led to the decrease in the initial grain size of SiC_p_/Mg-5Zn material, as shown in Figure 1. The grain size has an important influence on twin nucleation. The critical shear stress (CRSS) required for the formation of twins increases with the decrease in the grain size of Mg alloys [22,23]. Therefore, no twins were found in the SiC_p_/Mg-5Zn material when the compression strain was ~0.05 due to the combined effect of the above two aspects.

**Figure 6:**
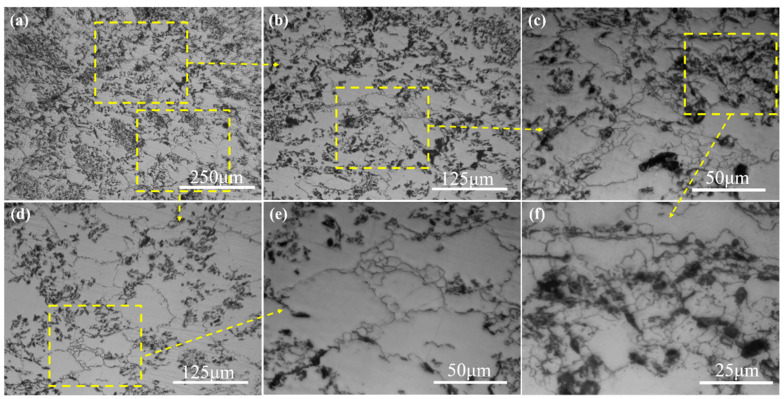
OM microstructure of the SiC_p_/Mg-5Zn material with a compression strain of 0.1: (**a**,**b**,**d**) low magnification and (**c**,**e**,**f**) high magnification.

As the compression strain increased to ~0.1 (as shown in Figure 6), it can be seen that more DRXed grains were formed at the original grain boundaries in SiC_p_/Mg-5Zn material, and the average DRXed grain size and DRXed ratio were ~2.9 μm and ~23.6%, respectively. It can be concluded that the volume fraction of DRXed grains in SiC_p_/Mg-5Zn material increased with the increment of the compression strain from ~0.05 to ~0.1, but the average DRXed grain size decreased.

**Figure 7:**
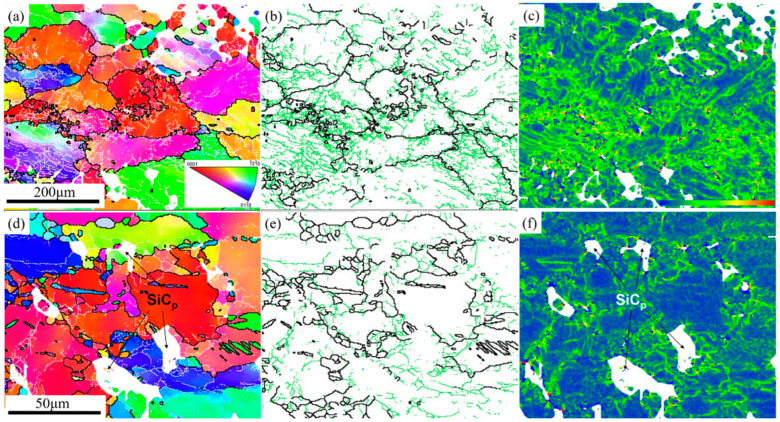
EBSD results of the SiC_p_/Mg-5Zn material with a compression strain of 0.1: (**a**,**d**) IPF maps, (**b**,**e**) grain boundaries distribution maps and (**c**,**f**) KAM map.

Figure 7 shows the EBSD results of the SiC_p_/Mg-5Zn material with a compression strain of ~0.1. It can be seen from the IPF maps that the grain boundaries had an obvious bending phenomenon, and fine DRXed grains can also be seen at the original grain boundaries, as shown in Figure 7. This result is consistent with the OM microstructure. 

Figure 7b,e shows the grain boundary distribution maps of the SiC_p_/Mg-5Zn material. The green line is the grain boundary with low angle, and its misorientation angle was greater than 2° but less than 15°; the black line represents the large angle grain boundary with a misorientation angle greater than 15°. It can be seen that the original grain boundary of the SiC_p_/Mg-5Zn material was the high angle grain boundary represented by black lines, and the original grain boundary bent after compression, resulting in a “bulge”. Meanwhile, the fine DRXed grains were observed at some of the original grain boundaries, which further confirms that the bulging nucleation of grain boundaries is a nucleation mechanism of the SiC_p_/Mg-5Zn material.

Moreover, the KAM maps of SiC_p_/Mg-5Zn material with the compression strain of ~0.1 are given in Figure 7c,f. It can be seen that a large distortion was produced in some grains after compression (green area). The difference of strain energy storage in different grains was mainly due to the inhomogeneity of deformation. Meanwhile, it can be seen from Figure 7c,f that an obvious lattice distortion was also formed around SiC_p_, and the strain storage energy was generated.

**Figure 8:**
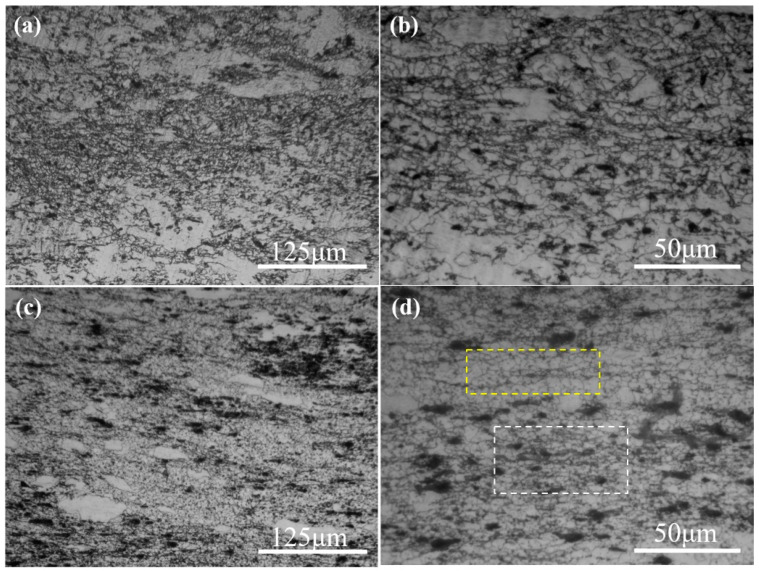
OM microstructures of the SiC_p_/Mg-5Zn material with compression strains of (**a**,**b**) 0.35 and (**c**,**d**) 0.7, the yellow box in (**d**) is the area away from particles and the white box in (**d**) is the area near the particles.

**Figure 9:**
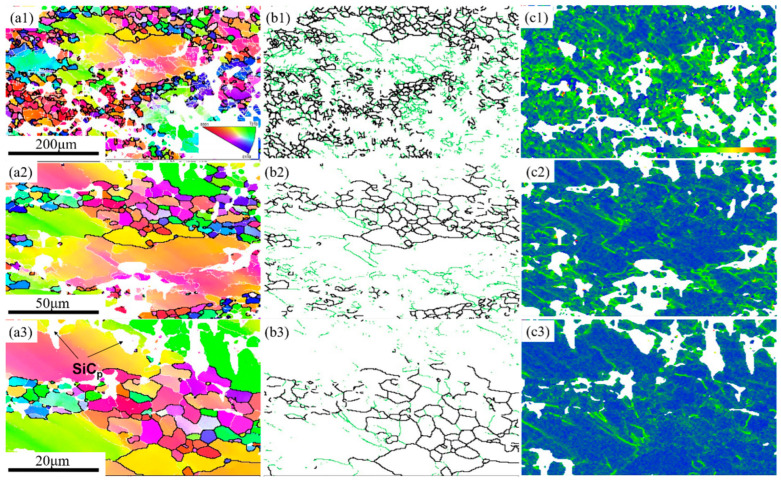
EBSD results of the SiC_p_/Mg-5Zn material with a compression strain of 0.35: (**a1**–**a3**) IPF maps, (**b1**–**b3**) grain boundary distribution maps and (**c1**–**c3**) KAM map.

When the compression strain improved to ~0.35, the DRXed ratio in SiC_p_/Mg-5Zn material increased obviously, and the deformed grains disappeared, as shown in Figure 8a,b. Meanwhile, the SiC_p_/Mg-5Zn material with a compression strain of ~0.35 was characterized by EBSD, as shown in Figure 9. It can be seen that DRXed ratio in SiC_p_/Mg-5Zn material increased significantly with the improvement in the compression strain from ~0.1 to ~0.35, which is consistent with the OM microstructure (Figure 6 and Figure 8a,b). However, no fine DRXed grains were observed near SiC_p_, as shown in Figure 9a2,a3.

**Figure 10:**
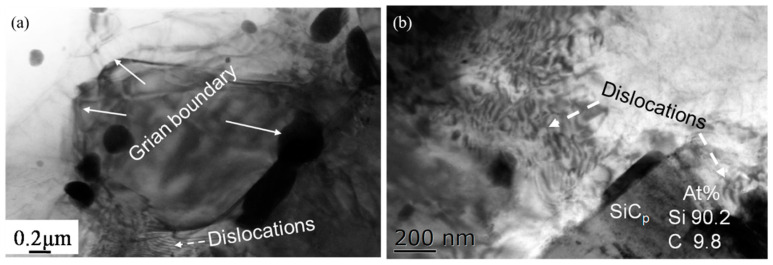
TEM microstructure of the SiC_p_/Mg-5Zn material with a compression strain of 0.35.

Figure 10 shows the TEM microstructure of the SiC_p_/Mg-5Zn material with a compression strain of ~0.35. It can be seen from Figure 10a that the DRXed grain was formed in the SiC_p_/Mg-5Zn material after compression, and the secondary phase was distributed along the DRXed grain boundary. Meanwhile, high density dislocations were found around SiC_p_, as shown in Figure 10b. A large number of dislocations were generated around the particles due to the deformation mismatch between the particles and matrix during deformation [13].

As the compression strain increased to ~0.7, the SiC_p_/Mg-5Zn material almost underwent complete DRX, as shown in Figure 8c,d. The average DRXed grain size and volume fraction of SiC_p_/Mg-5Zn were ~2.8 μm and ~97.8%. In addition, it can be seen from Figure 8d that there were obvious DRXed grains near SiC_p_ when the compression strain was ~0.7, and the size of DRXed grains around SiC_p_ (in the white box) was smaller than that of the DRXed grains far away from SiC_p_ (in yellow box), indicating that fine DRXed grains were formed around the particles due to the PDZ effect when the compression strain increased to ~0.7 [13].

### 3.3. Precipitation Behavior

**Figure 11:**
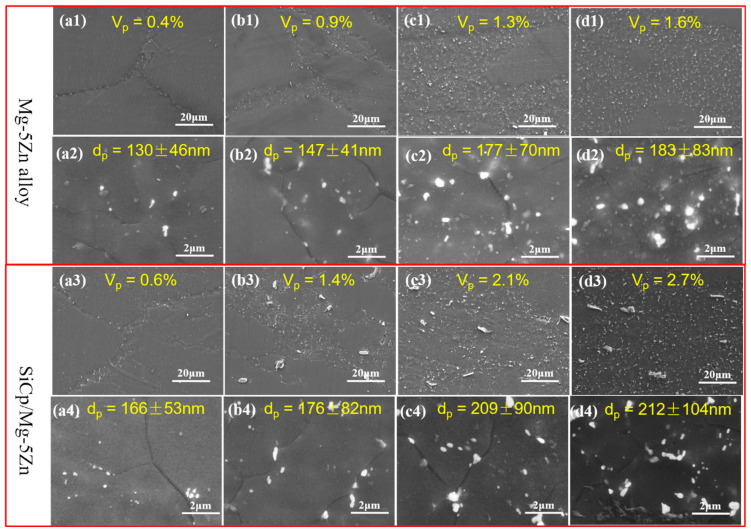
SEM microstructures of the Mg-5Zn alloy and the SiC_p_/Mg-5Zn material with different compression strains: (**a1**–**a4**) 0.05, (**b1**–**b4**) 0.1, (**c1**–**c4**) 0.35 and (**d1**–**d4**) 0.7.

**Figure 12:**
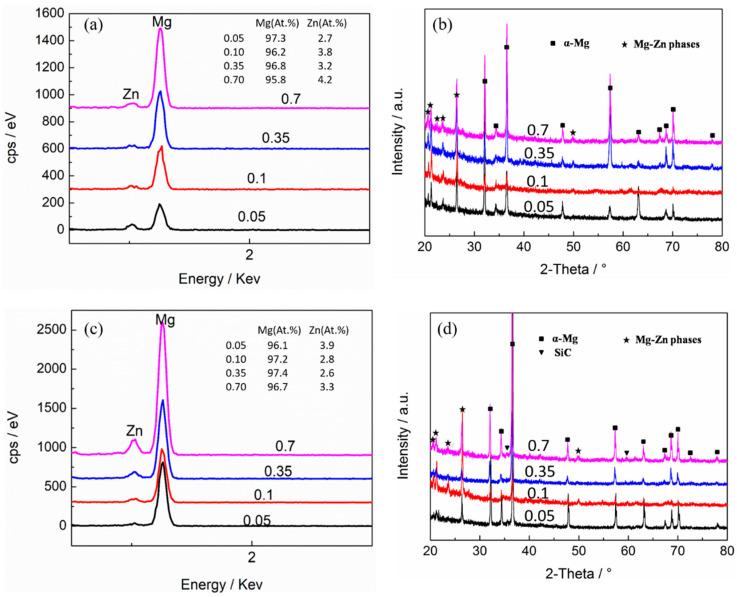
(**a**,**c**) EDS and (**b**,**d**) XRD of (**a**,**b**) the Mg-5Zn alloy and (**c**,**d**) the SiC_p_/Mg-5Zn material with different compression strains.

Figure 11 shows the SEM microstructure of the as-compressed Mg-5Zn alloy and SiC_p_/Mg-5Zn material. It can be seen that the white secondary phases precipitated in the Mg-5Zn alloy when the compression strain was ~0.05, as shown in Figure 11a1,a2. The EDS and XRD results show that the white precipitated phases were Mg-Zn phases, as shown in Figure 12a,b. Moreover, it can be found that the Mg-Zn phases were distributed along the grain boundaries. 

Generally, the grain boundaries can be used as a favorable location for the nucleation of the secondary phase due to the high density of dislocations and vacancies [16]. Furthermore, the dislocation can provide a channel for Zn atom diffusion, which is conducive to the nucleation and growth of the secondary phases [8]. While the dislocation density at the grain boundaries was high, due to the movement of dislocation it was hindered by the grain boundaries. Therefore, the secondary phases in the Mg-5Zn alloy mainly precipitated along the grain boundaries. The average size and volume fraction of the Mg-Zn phases in Mg-5Zn alloy were ~130 nm and ~0.4% when the compression strain was ~0.05.

The average size and volume fraction of Mg-Zn phases in the Mg-5Zn alloy increased to ~147 nm and~0.9% when the compression strain improved from ~0.05 to ~0.1, as shown in Figure 11b1,b2. This reason can be attributed to the enhancement of the DRXed ratio for the Mg-5Zn alloy with the increment of the compression strain (Figure 2), resulting in an increase in the amount of the grain boundaries, which can provide more nucleation sites for the dynamic precipitation of the secondary phases.

**Figure 13:**
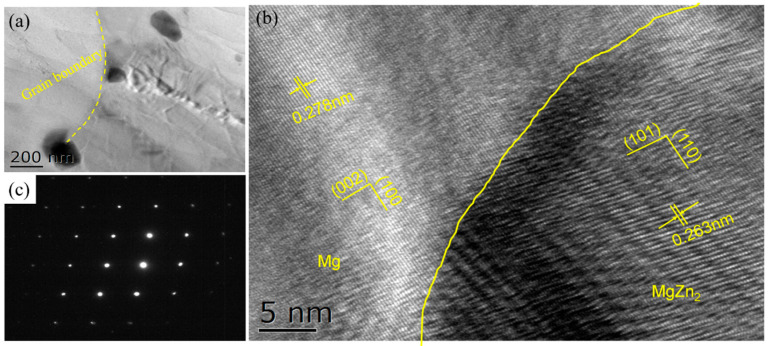
(**a**) Bright field image, (**b**) HRTEM map and (**c**) diffraction pattern of the precipitate in the Mg-5Zn alloy with a compression strain of 0.1.

Figure 13 gives the TEM microstructure of the Mg-5Zn alloy with a compression strain of ~0.1. It can be seen that the Mg-Zn phase was mainly distributed along the grain boundary (Figure 13a); this result is consistent with SEM. Further combining with the selected diffraction analysis results, the precipitated phase in the Mg-5Zn alloy can be identified as the MgZn_2_ phase. In addition, the interface relationship between the MgZn_2_ phase and the Mg matrix can be obtained through HRTEM analysis. It can be seen that the (110) plane of MgZn_2_ phase was parallel to the (100) plane of Mg ((110)_MgZn2_//(100)_Mg_), as shown in Figure 13b, in which, the plane distance of (110) of MgZn_2_ was ~0.263 nm, and the plane distance of (100) of Mg was ~0.278 nm. Therefore, the interface mismatch between MgZn_2_ and Mg matrix can be calculated as ~5.3% by Equation (1) [18], indicating that the interface bonding was good.

(1) $$\delta =(d{\left(100\right)}_{\mathrm{Mg}}-d{\left(110\right)}_{\mathrm{MgZn}2})/d{\left(100\right)}_{\mathrm{Mg}}\times 100\%$$

When the compression strain increased to ~0.35 and ~0.7, the improvement of the energy storage in Mg-5Zn alloy promoted the occurrence of DRX, which further increased the number of the grain boundaries. Thus, the size and volume fraction of the secondary phase in Mg-5Zn alloy increased with the increment of the compression strain. The average size of Mg-Zn phase was ~177 nm and ~183 nm, respectively, and the volume fraction was ~1.3% and ~1.6%, when the compression strain was ~0.35 and ~0.7.

**Figure 14:**
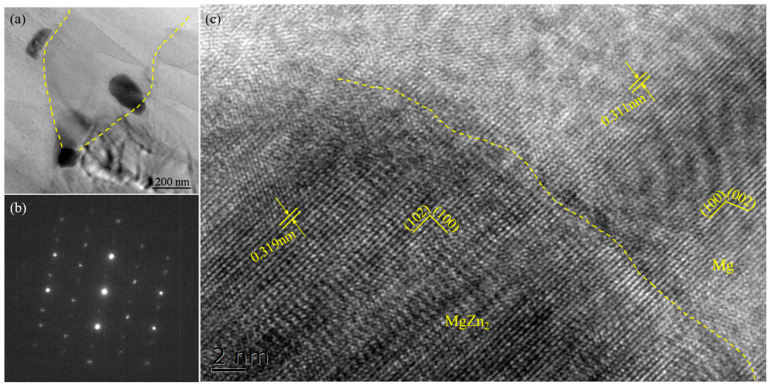
(**a**) TEM morphology, (**b**) HRTEM map and (**c**) diffraction pattern of the precipitate in the SiC_p_/Mg-5Zn material with a compression strain of 0.1.

Similar to the Mg-5Zn alloy, the SiC_p_/Mg-5Zn material also had an obvious secondary phase after compression, and the secondary phase was also distributed along the grain boundaries, as shown in Figure 11. Moreover, the secondary phase in SiC_p_/Mg-5Zn material was also determined to be the MgZn_2_ phase according to the results of EDS, XRD and TEM, as shown in Figure 12c,d and Figure 14.

HRTEM analysis showed that the (102) plane of MgZn_2_ phase was parallel to the (100) plane of Mg, as shown in Figure 14c. The plane distance of (102) of MgZn_2_ was ~0.319 nm, and the plane distance of (100) of Mg was ~0.311 nm. Thus, the interface mismatch between MgZn_2_ and Mg matrix can be calculated as ~2.5% by Equation (2) [18], which indicates that the interface between the MgZn_2_ phase and the Mg matrix was well bonded and had a coherent interface relationship.

(2) $$\delta =(d{\left(100\right)}_{\mathrm{Mg}}-d{\left(102\right)}_{\mathrm{MgZn}2})/d{\left(100\right)}_{\mathrm{Mg}}\times 100\%$$

**Figure 15:**
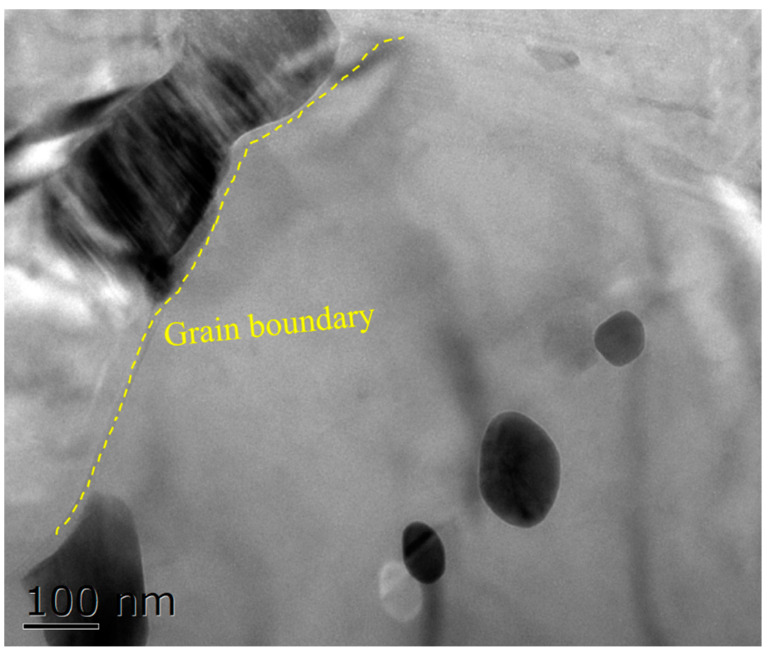
TEM microstructure of the SiC_p_/Mg-5Zn material with a compression strain of 0.35.

As the compression strain increased to ~0.35, it was found from Figure 11c3,c4 that some smaller MgZn_2_ phases appeared in the grains of the SiC_p_/Mg-5Zn material except for the distribution along the grain boundaries. Meanwhile, it can also be seen from the TEM microstructure that the size of MgZn_2_ phase dynamically precipitated in the grain of SiC_p_/Mg-5Zn material was obviously finer than that of the MgZn_2_ phase distributed along the grain boundary, as shown in Figure 15. It shows that the grain boundary not only provides the nucleation site for MgZn_2_ phase, but also promotes the growth of the MgZn_2_ phase.

Compared with the secondary phase in Mg-5Zn alloy, it can be seen that the volume fraction and average size of MgZn_2_ phase in SiC_p_/Mg-5Zn material were both greater than those in the Mg-5Zn alloy under the same compression strain (Figure 11), indicating that the addition of SiC_p_ promotes the precipitation and growth of the secondary phase in the Mg-5Zn matrix.

### 3.4. Texture Distribution

**Figure 16:**
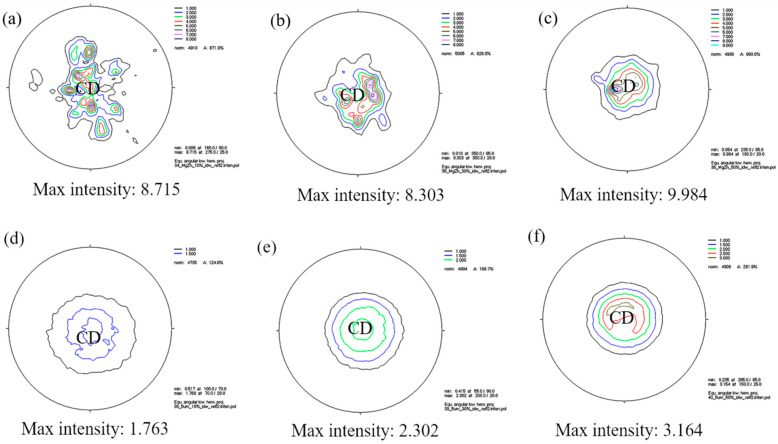
Macro-texture distribution of (0002) basal plane of (**a**–**c**) Mg-5Zn alloy and (**d**–**f**) SiC_p_/Mg-5Zn material with different compression strains: (**a**,**d**) 0.1, (**b**,**e**) 0.35 and (**c**,**f**) 0.7.

In order to study the influence of SiC_p_ on the texture of Mg-5Zn alloy, the (0002) plane macro-texture distribution of Mg-5Zn alloy and the SiC_p_/Mg-5Zn material was measured by neutron diffraction. Figure 16 shows the texture distribution of the as-compressed Mg-5Zn alloy and SiC_p_/Mg-5Zn material. It can be seen that a typical (0002) basal plane texture was formed for the Mg-5Zn alloy and the SiC_p_/Mg-5Zn material after compression. In other words, the (0002) basal plane was perpendicular to the compression direction (CD). 

Moreover, it can be seen form Figure 16 that the compression strain had little influence on the texture type but had significant influence on the maximum intensity. The maximum intensity of the (0002) basal plane texture was 8.715 mrd (mrd: multiples of random distribution) for the Mg-5Zn alloy when the compression strain was ~0.1, as shown in Figure 16a. The maximum intensity decreased to 8.303 mrd when the compression strain improved from ~0.1 to ~0.35.

However, the maximum intensity of (0002) basal plane texture of Mg-5Zn alloy did not decrease monotonously with the increment of compression strain. When the compression strain of Mg-5Zn alloy increased from ~0.35 to ~0.7, the change of texture type of alloy was still small, but the maximum intensity increased from 8.303 mrd to 9.984 mrd, as shown in Figure 16c.

Compared with Mg-5Zn alloy, the texture type of SiC_p_/Mg-5Zn material did not change, but the maximum intensity of the (0002) basal plane texture of SiC_p_/Mg-5Zn material was less than that of Mg-5Zn alloy under the same compression strain. Meanwhile, it can be seen that, unlike the Mg-5Zn alloy, the maximum intensity of the basal plane texture for SiC_p_/Mg-5Zn increased monotonously with the improvement in the compression strain, as shown in Figure 16d–f.

The above research shows that the introduction of SiC_p_ can weaken the basal texture strength of Mg-5Zn matrix, which is mainly due to: on the one hand, SiC_p_ itself can change the original flow direction during the deformation, thereby weakening the texture; on the other hand, the volume fraction of DRXed grains in SiC_p_/Mg-5Zn material is higher than that in the Mg-5Zn alloy, and DRXed grains have a more random orientation, which can weaken the texture of materials [24].

In addition, the presence of precipitates in Mg alloys can also weaken the texture [25,26]. First, the precipitates can be used as DRX nucleation sites, which increase the volume fraction of DRXed grains and weaken the texture; second, precipitates are often generated at the grain boundary, which has a certain pinning effect on the rotation of the grain boundary, leading to texture weakening [27,28]. Therefore, the texture of the material can be weakened with the increment of the volume fraction of precipitates. According to the statistical results of the secondary phase after compression of Mg-5Zn alloy and SiC_p_/Mg-5Zn material, the addition of SiC_p_ can enhance the volume fraction of the secondary phase in Mg-5Zn alloy, as shown in Figure 11. Thus, the maximum intensity of the basal texture of SiC_p_/Mg-5Zn material was less than that of Mg-5Zn alloy due to the combined effect of the above aspects.

Furthermore, the maximum intensity of the basal texture of Mg-5Zn alloy does not decrease monotonously with the increase of compression strain, but first decreases and then increases. This was attributed to the formation of (10$\bar{1}$2) tensile twin when the compression strain was ~0.1, as shown in Figure 2b1,b2. Hong et al. [29,30] found that the appearance of (10$\bar{1}$2) tensile twin in Mg alloys can lead to the enhancement of maximum intensity for basal texture. Hence, the maximum intensity of the basal texture for Mg-5Zn alloys is larger at the initial stage of compression (compression strain of ~0.1). However, the volume fraction of DRXed grains in the Mg-5Zn alloy increased, and the number of twins decreased significantly because twins promoted DRX nucleation when the compression strain improved from ~0.1 to ~0.35, as shown in Figure 2c1,c2. Therefore, the maximum intensity of the basal texture for Mg-5Zn alloy decreased as the compression strain increased from ~0.1 to ~0.35.

## 4. Discussion

### 4.1. Effect of SiC_p_ on DRX Behavior

According to the analysis of the microstructure evolution of the Mg-5Zn alloy and the SiC_p_/Mg-5Zn material during hot compression, it can be seen that the addition of SiC_p_ improved the DRXed ratio of Mg-5Zn alloy, as shown in Figure 2, Figure 5, Figure 6 and Figure 8. Meanwhile, the DRXed ratio in both Mg-5Zn alloy and SiC_p_/Mg-5Zn material increased with the increment of the compression strain.

**Figure 17:**
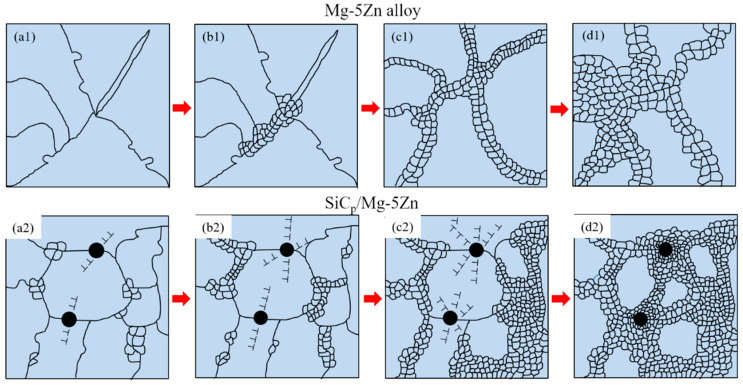
Schematic diagrams of DRX for the Mg-5Zn alloy and SiC_p_/Mg-5Zn material with different compression strains: (**a1**,**a2**) 0.05, (**b1**,**b2**) 0.1, (**c1**,**c2**) 0.35 and (**d1**,**d2**) 0.7.

Based on the above research results, the diagram of microstructure evolution of Mg-5Zn alloy and SiC_p_/Mg-5Zn material during hot compression is shown in Figure 17. There was no obvious DRXed grains in Mg-5Zn alloy when the compression strain was ~0.05, but the grain boundary appeared to have obvious “bulging” phenomenon and twins were formed in the alloy, as shown in Figure 17a. 

With the increase in the compression strain to ~0.1, fine DRXed grains were formed at the original grain boundary of Mg-5Zn alloy, especially at the intersection of grain boundary and twin, as shown in Figure 17b. The volume fraction and average grain size of DRXed grains in Mg-5Zn alloy increased significantly when the compression strain increased to ~0.35, and the twin structure was engulfed by fine DRXed grains, and the number of twins decreased gradually. 

The PDZ (particle deformation zone) with high-density dislocations was formed around the particles at the initial stage of compression for the SiC_p_/Mg-5Zn material due to the deformation mismatch between the particle and Mg matrix. Moreover, the grain boundary also provided favorable conditions for DRX nucleation, since the movement of dislocations was hindered by the grain boundary, which led to the stacking of dislocations at the grain boundary. However, the effect of PDZ on DRX nucleation was related to the compression strain. The promotion effect of PDZ on DRX nucleation increasesd with the increment of compression strain. The DRXed grains in SiC_p_/Mg-5Zn material mainly nucleated along the grain boundary when the compression strain (~0.05, ~0.1 and ~0.35) was small, as shown in Figure 17a2,b2,c2. As the compression strain of SiC_p_/Mg-5Zn material improved to ~0.7, the fine recrystallized grains also appeared around the particles due to the PDZ effect, in addition to the nucleation at the grain boundary (Figure 17d2).

This may be due to the fact that the dislocation density in the PDZ around the particles in SiC_p_/Mg-5Zn material was low and the misorientation gradient was poor when the compression strain was small, which was not enough to stimulate the nucleation of DRXed grains. Therefore, its recrystallization mechanism is similar to that of the Mg-5Zn alloy and the grain boundary is the main nucleation location of DRXed grains. Nevertheless, the addition of SiC_p_ can reduce the original grain size of SiC_p_/Mg-5Zn material and increase the number of grain boundaries, so the DRXed ratio of SiC_p_/Mg-5Zn material was also greater than that of the Mg-5Zn alloy at the initial stage of the compression strain (~0.05, ~0.1 and ~0.35). However, the DRXed grains were formed around the particles due to the PSN effect when the compression strain increased to ~0.7, which further improved the DRXed ratio of the SiC_p_/Mg-5Zn material.

### 4.2. Effect of SiC_p_ on Precipitation Behavior

It can be seen from Figure 11 that the size and volume fraction of dynamic precipitated phase in the Mg-5Zn alloy and SiC_p_/Mg-5Zn material increased with the increment of compression strain, indicating that the secondary phase in the Mg-5Zn alloy and SiC_p_/Mg-5Zn material continuously precipitated and grew during hot compression.

Compared with the Mg-5Zn alloy, the size and volume fraction of the precipitated phase in the SiC_p_/Mg-5Zn material were both higher than that in Mg-5Zn alloy under the same compression strain. It can be seen that the introduction of SiC_p_ can promote the nucleation and growth of precipitates. The influence of SiC_p_ on the dynamic precipitation behavior of the secondary phase can be attributed to the effect of SiC_p_ on the DRX behavior of Mg alloy matrix [16].

It is generally accepted that the dynamic precipitation of the secondary phase mainly includes two stages of phase nucleation and growth. According to the thermodynamic of precipitation, the decrease in Gibbs free energy of matrix can provide driving force for the nucleation of precipitates, however, the interface energy and mismatch between matrix and precipitates will hinder the formation of precipitates [8]. Therefore, the positions where the interface energy and strain energy decrease are conducive to the formation of precipitates, such as vacancy group, dislocation and grain boundary [31,32]. Due to the high density of dislocations and vacancies at the grain boundary, the energy is high, which helps the nucleation of precipitated phases [33]. The diffusion rate of atoms also plays an important role in the dynamic precipitation behavior of materials during hot deformation. Generally, plastic deformation can generate a large number of dislocations and vacancies in the material, which provides a favorable channel for atomic diffusion [27].

The PDZ with high density dislocation and large misorientation gradient was formed around particles in the SiC_p_/Mg-5Zn material after the compression (Figure 17), which was conducive to the diffusion of Zn atoms, thus promoting the nucleation of the Mg-Zn phase [8]. Meanwhile, compared with dislocation and vacancy, the diffusion rate of atoms at the grain boundary was higher. The DRX region contained more grain boundaries, which can provide more favorable nucleation sites for the dynamic precipitation of the secondary phase, so the secondary phase often precipitated in the DRX region. Li et al. [8] found a large number of the secondary phase are mainly distributed in the DRX region, while only a small amount of precipitation phase existed in the grain, which indicated that the grain boundary created more favorable conditions for the nucleation of the secondary phase.

By comparing the volume fraction of DRXed grains in Mg-5Zn alloy and SiC_p_/Mg-5Zn material during compression, it can be seen that the volume fraction of DRXed grains in SiC_p_/Mg-5Zn material was greater than that in the Mg-5Zn alloy at the same strain. Therefore, more favorable nucleation sites for the dynamic precipitation of the secondary phase were provided for the SiC_p_/Mg-5Zn material due to the addition of SiC_p_ which increased the number of grain boundaries. Then, the volume fraction of secondary phase in SiC_p_/Mg-5Zn material was greater than that in the Mg-5Zn alloy.

In addition, the size of precipitated phase in SiC_p_/Mg-5Zn material was larger than that in the Mg-5Zn alloy, which was mainly related to the DRXed grain size and DRXed ratio. As previously mentioned, the DRXed grain size of SiC_p_/Mg-5Zn material was smaller than that of the Mg-5Zn alloy, and the DRXed ratio in SiC_p_/Mg-5Zn material was greater than that of the Mg-5Zn alloy, thus increasing the number of diffusion channels of Zn atoms in SiC_p_/Mg-5Zn material, leading to the increase in growth rate and size of precipitates.

## 5. Conclusions

In this paper, the Mg-5Zn alloy and SiC_p_/Mg-5Zn material were compressed by using a thermal simulator, and the microstructure evolution of the Mg-5Zn alloy and SiC_p_/Mg-5Zn material was studied under the different compression strains. The following conclusions are summarized as follows:

(1) DRXed grains were mainly formed in the Mg-5Zn alloy by the “bulging” nucleation mechanism of the original grain boundary during compression. Moreover, the formation of twins also induced the formation of DRXed grains. The volume fraction and average DRXed grain size of Mg-5Zn alloy increased with the increment of compression strain;
(2) The introduction of SiC_p_ improved the DRXed ratio of the Mg-5Zn matrix, but DRXed grains in SiC_p_/Mg-5Zn material were still mainly formed by the mechanism of “bulging” nucleation under low strain. However, there were obviously DRXed grains around the particles under higher strain, and the DRXed grains near the particles were smaller than those far away from the particles, which was credited to the PSN effect of particle;
(3) The addition of SiC_p_ enhanced the volume fraction and size of precipitates in the Mg-5Zn alloy. Meanwhile, the addition of SiC_p_ had little effect on the texture type of the Mg-5Zn alloy but decreased the maximum intensity of (0002) basal texture.

## Data Availability

The data that support the findings of this study are available from the corresponding author upon reasonable request.

## References

[1] Nie J.F. (2011). Precipitation and Hardening in Magnesium Alloys. Metall. Mater. Trans. A.

[2] Wang B., Xu D., Wang S., Sheng L., Zeng R.-C., Han E.-H. (2019). Influence of solution treatment on the corrosion fatigue behavior of an as-forged Mg-Zn-Y-Zr alloy. Int. J. Fatigue.

[3] Li Y., Hou P., Wu Z., Feng Z., Ren Y., Choo H. (2021). Dynamic recrystallization of a wrought magnesium alloy: Grain size and texture maps and their application for mechanical behavior predictions. Mater. Des..

[4] Pan H., Kang R., Li J., Xie H., Zeng Z., Huang Q., Yang C., Ren Y., Qin G. (2020). Mechanistic investigation of a low-alloy Mg-Ca based extrusion alloy with high strength-ductility synergy. Acta Mater..

[5] Estrin Y., Vinogradov A. (2013). Extreme grain refinement by severe plastic deformation: A wealth of challenging science. Acta Mater..

[6] Derazkola H.A., García Gil E., Murillo-Marrodán A., Méresse D. (2021). Review on Dynamic Recrystallization of Martensitic Stainless Steels during Hot Deformation: Part I—Experimental Study. Metals.

[7] El-Sayed M.M., Shash A., Abd-Rabou M., ElSherbiny M.G. (2021). Welding and processing of metallic materials by using friction stir technique: A review. J. Adv. Join. Process..

[8] Li W.J., Deng K.K., Zhang X., Wang C.J., Kang J.-W., Nie K.-B., Liang W. (2017). Microstructures, tensile properties and work hardening behavior of SiCp/Mg-Zn-Ca composites. J. Alloy. Compd..

[9] Shi Q.-X., Deng K.-K., Nie K.-B., Zhang W.-G., Cao M., Liang W. (2020). Significant Influence of Minor SiCp on Microstructure and Mechanical Properties of Pure Mg. J. Mater. Eng. Perform..

[10] Nie K.-B., Zhu Z.-H., Munroe P., Deng K.-K., Guo Y.-C. (2020). Microstructure and mechanical properties of TiC nanoparticle-reinforced Mg-Zn-Ca matrix nanocomposites processed by combining multidirectional forging and extrusion. Trans. Nonferrous Met. Soc. China.

[11] Sun X.-F., Wang C.-J., Deng K.-K., Nie K.-B., Zhang X.-C., Xiao X.-Y. (2018). High strength SiCp/AZ91 composite assisted by dynamic precipitated Mg 17 Al 12 phase. J. Alloy. Compd..

[12] Wang X.-J., Hu X.-S., Nie K.-B., Wu K., Zheng M.-Y. (2012). Hot extrusion of SiCp/AZ91 Mg matrix composites. Trans. Nonferrous Met. Soc. China.

[13] Doherty R., Hughes D., Humphreys F., Jonas J., Jensen D., Kassner M., King W., McNelley T., McQueen H., Rollett A. (1997). Current issues in recrystallization: A review. Mater. Sci. Eng. A.

[14] Wu K., Deng K., Nie K.B., Wu Y., Wang X., Hu X., Zheng M. (2010). Microstructure and mechanical properties of SiCp/AZ91 composite deformed through a combination of forging and extrusion process. Mater. Des..

[15] Wang X.J., Hu X.S., Nie K.B., Deng K.K., Wu K., Zheng M.Y. (2012). Dynamic recrystallization behavior of particle reinforced Mg matrix composites fabricated by stir casting. Mater. Sci. Eng. A.

[16] Shi Q.-X., Wang C.-J., Deng K.-K., Nie K.-B., Wu Y., Gan W.-M., Liang W. (2021). Microstructure and mechanical behavior of Mg-5Zn matrix influenced by particle deformation zone. J. Mater. Sci. Technol..

[17] Shen J., Zhang L., Hu L., Sun Y., Gao F., Liu W., Yu H. (2021). Effect of subgrain and the associated DRX behaviour on the texture modification of Mg-6.63Zn-0.56Zr alloy during hot tensile deformation. Mater. Sci. Eng. A.

[18] Hu G.X., Cai X., Rong Y.H. (2010). Fundamentals of Materials Science.

[19] Xu S., Kamado S., Matsumoto N., Honma T., Kojima Y. (2009). Recrystallization mechanism of as-cast AZ91 magnesium alloy during hot compressive deformation. Mater. Sci. Eng. A.

[20] Nie H., Hao X., Kang X., Chen H., Chi C., Liang W. (2020). Strength and plasticity improvement of AZ31 sheet by pre-inducing large volume fraction of (10–12) tensile twins. Mater. Sci. Eng. A.

[21] Liu X., Zhu B., Xie C., Zhang J., Tang C., Chen Y. (2018). Twinning, dynamic recrystallization, and crack in AZ31 magnesium alloy during high strain rate plane strain compression across a wide temperature. Mater. Sci. Eng. A.

[22] Liu T., Yang Q., Guo N., Lu Y., Song B. (2020). Stability of twins in Mg alloys—A short review. J. Magn. Alloy..

[23] Zhao T.S., Hu Y.B., Zhang C., He B., Zheng T.X., Tang A.T., Pan F.S. (2022). Influence of extrusion conditions on microstructure and mechanical properties of Mg-2Gd-0.3Zr magnesium alloy. J. Magn. Alloy..

[24] Tong L.B., Zheng M.Y., Cheng L.R., Kamado S., Zhang H.J. (2013). Effect of extrusion ratio on microstructure, texture and mechanical properties of indirectly extruded Mg–Zn–Ca alloy. Mater. Sci. Eng. A.

[25] Chen C., Chen J., Yan H., Su B., Song M., Zhu S. (2016). Dynamic precipitation, microstructure and mechanical properties of Mg-5Zn-1Mn alloy sheets prepared by high strain-rate rolling. Mater. Des..

[26] Shi Q.-X., Wang C.-J., Deng K.-K., Nie K.-B., Cao M., Gan W.-M., Liang W. (2020). Work hardening and softening behavior of pure Mg influenced by Zn addition investigated via in-situ neutron diffraction. Mater. Sci. Eng. A.

[27] Li W.-J., Deng K.-K., Zhang X., Nie K.-B., Xu F.-J. (2016). Effect of ultra-slow extrusion speed on the microstructure and mechanical properties of Mg-4Zn-0.5Ca alloy. Mater. Sci. Eng. A.

[28] Du Y.Z., Qiao X.G., Zheng M.Y., Wu K., Xu S.W. (2015). The microstructure, texture and mechanical properties of extruded Mg-5.3Zn-0.2Ca-0.5Ce (wt%) alloy. Mater. Sci. Eng. A.

[29] Hong S.G., Park S.H., Chong S.L. (2010). Role of {10–12} twinning characteristics in the deformation behavior of a polycrystalline magnesium alloy. Acta Mater..

[30] Hong S.G., Park S.H., Chong S.L. (2011). Strain path dependence of {10–12} twinning activity in a polycrystalline magnesium alloy. Scr. Mater..

[31] Mendis C., Hono K. (2013). Understanding precipitation processes in magnesium alloys. Fundament. Magnes. Alloy. Metall..

[32] Guo F., Zhang D., Yang X., Jiang L., Pan F. (2015). Strain-induced dynamic precipitation of Mg17Al12 phases in Mg–8Al alloys sheets rolled at 748K. Mater. Sci. Eng. A.

[33] Kabir A.S.H., Sanjari M., Su J., Jung I.-H., Yue S. (2014). Effect of strain-induced precipitation on dynamic recrystallization in Mg–Al–Sn alloys. Mater. Sci. Eng. A.

